# Cognitive behaviour therapy in medication-treated adults with ADHD and persistent Symptoms: A randomized controlled trial

**DOI:** 10.1186/1471-244X-11-116

**Published:** 2011-07-25

**Authors:** Brynjar Emilsson, Gisli Gudjonsson, Jon F Sigurdsson, Gisli Baldursson, Emil Einarsson, Halldora Olafsdottir, Susan Young

**Affiliations:** 1 King's College London, Institute of Psychiatry, De Crespigny Park, London, UK; 2 Mental Health Services, Landspitali - The National University Hospital of Iceland, Hringbraut, Reykjavik, Iceland; 3 Child- and Adolescent Psychiatry, Landspitali - The National University Hospital of Iceland, Dalbraut 12, Reykjavik, Iceland

## Abstract

**Background:**

Attention deficit hyperactivity disorder (ADHD) in adulthood is not fully treated by psychopharmacological treatment alone. The main aim of the current study was to evaluate a newly developed cognitive behaviour therapy (CBT) based group programme, the Reasoning and Rehabilitation for ADHD Youths and Adults (R&R2ADHD), using a randomized controlled trial.

**Methods:**

54 adults with ADHD already receiving psychopharmacological treatment were randomly allocated to an experimental (CBT/MED) treatment condition (n = 27) and a 'treatment as usual' (TAU/MED) control condition (n = 27) that did not receive the CBT intervention. The outcome measures were obtained before treatment (baseline), after treatment and at three month follow-up and included ADHD symptoms and impairments rated by independent assessors, self-reported current ADHD symptoms, and comorbid problems.

**Results:**

The findings suggested medium to large treatment effects for ADHD symptoms, which increased further at three month follow-up. Additionally, comorbid problems also improved at follow-up with large effect sizes.

**Conclusions:**

The findings give support for the effectiveness of R&R2ADHD in reducing ADHD symptoms and comorbid problems, an improving functions associated with impairment. The implications are that the benefits of R&R2ADHD are multifaceted and that combined psychopharmacological and CBT based treatments may add to and improve pharmacological interventions.

**Trial registration:**

ACTRN12611000533998 (http://www.ANZCTR.org.au/ACTRN12611000533998.aspx)

## Background

In the last decade ADHD among adults has become increasingly recognized as a complex disorder characterized by high rates of comorbidity and social dysfunction, including mood disorders, anxiety, alcohol and drug abuse, educational failure, occupational problems, interpersonal relationship problems, delinquency and crime [1-4]. Population surveys estimate the prevalence of ADHD in adults to be around 2.5% [5].

Many adults do not obtain their diagnosis until their adult years yet even when ADHD has been recognized and treated in childhood psychiatric and psychosocial outcomes are bleak [6,7]. The costs associated with the disorder are serious and long-term [8].

In addition to high rates of comorbidity, adult ADHD has been associated with maladaptive personality (i.e. a disorganized personality style) and maladaptive coping strategies which limits the internal resources available to the individual [9,10]. Thus treatments need to not only target symptom reduction, but aim to improve quality of life by addressing the multiple problems that impair daily social and emotional functioning [11].

International guidelines [8,12] recommend multimodal treatment for adults with ADHD comprising of psychoeducation, pharmacotherapy and cognitive behaviour therapy (CBT). The need for non-pharmacological interventions is underpinned by the finding that some adults do not respond to drug treatment and those who do may only experience a partial response [13]. In the past few years prescribing has increased for treating ADHD [14], yet psychological treatments have not paralleled this growth [2,15].

Research on the effectiveness of psychopharmacological treatments in ADHD adults has been extensive compared with evaluations of psychological interventions. Only six randomised controlled studies have been published and these all report effectiveness of CBT interventions in medicated patients. CBT provided on an individual basis has been evaluated by Safren and colleagues [16] who randomly assigned 31 patients receiving medication to receive 15 sessions of CBT or treatment as usual. They found that combined medication and CBT had a greater effect for independent evaluator ratings of ADHD symptoms, impairment and depression and for self-reported ADHD symptoms and anxiety. They later conducted another study randomizing medicated patients to either 12 sessions of CBT or relaxation with educational support and found similar results for ADHD symptom reduction [17]. Importantly, improvements for those who responded to treatment were maintained at 12 month follow up. In a study of 29 adults with ADHD (medication not controlled for) comparing 10 sessions of individual CBT with 20 sessions of cognitive training (CT; training of attention, executive functions and working memory) and a control condition, a significant effect was only found for self-reported inattention. No effect was found on independent evaluations, or on independent and self-ratings for measures of ADHD symptoms, depression or quality of life [18].

Group interventions are attractive for clinical delivery as they are cost effective, thus group interventions were recommended by the National Institute for Health and Clinical Excellence [NICE] as the first line psychological treatment. Solanto and colleagues [19] evaluated a 12 session group CBT programme by randomly assigning 88 patients receiving medication to receive either CBT or supportive therapy. The CBT condition had lower treatment dropout and found significant effect for self-report, collateral report and independent evaluator ratings of inattention symptoms. No significant effect was found for comorbid problems (depression, anxiety and self-esteem) or for organization and planning skills. A similar pattern of outcome was reported by Hirvikoski and colleagues [20] who randomly assigned 51 medicated adults to 14 sessions of dialectical behaviour therapy (DBT) or a loosely structured discussion group. A significantly greater reduction in ADHD symptoms was self-reported at the end of DBT group treatment but no significant difference was found for comorbid depression, anxiety, sleep problems, stress or functional impairment. Stevenson et al., [21] randomized 43 medicated patients to an eight week cognitive remediation therapy (CRT) group programme or treatment as usual and found a significant effect for ADHD symptoms, organizational skills and reduction in feelings of anger for those who completed the programme. The group programme introduced the novel element of individual coaching sessions for participants between group sessions. The treatment gains for the CRT condition were maintained at one year follow up except for state anger.

The only non-randomized controlled study that has been reported indicated that CBT can be effective even when provided in intensive bursts. Bramham and colleagues [22] provided an intensive 3-day intervention (one day per month for 3 months) to medicated patients and compared outcome with waiting list controls. The intervention included psychoeducation and CBT drawing on modules from the Young-Bramham programme [23] on topics of ADHD symptoms, emotional control, relationship skills, time-management, problem solving, and preparing for the future. A significant effect was found for those receiving CBT on measures of psychoeducation (an ADHD knowledge quiz), self-efficacy and self-esteem. No significant effect was found for anxiety and depression.

The findings from these studies suggest that the provision of psychological treatment in medicated patients - whether delivered in individual or group sessions - is effective in treating ADHD symptoms and has an additive effect over and above medication alone. The findings for treating comorbid problems however are limited and need to be studied further. Nevertheless comorbidity in adult ADHD is so common that group interventions that target symptoms, comorbid and associated problems will have a better chance of conferring health gain by making global improvements to self-efficacy, self-esteem and quality of life. If this can be achieved, this will be a cost-effective intervention that may reduce multiple presentations to health care services [6,24].

This study aimed to investigate the effectiveness of the R&R2 ADHD cognitive behavioural group treatment which has been developed to treat ADHD symptoms and common comorbid problems. Medicated patients were randomly assigned to either receive CBT (the CBT/MED condition) or treatment as usual (TAU/MED condition). The primary outcomes of interest were changes in ADHD symptoms following treatment. Secondary outcome measures were anxiety, depression, emotional control, social functioning and antisocial behaviour. It was hypothesized that the CBT/MED condition would show significantly greater improvements than the TAU/MED condition on primary and secondary outcome measures and that this effect would be maintained at follow-up.

## Methods

### Participants

Participants had been referred to an outpatient rehabilitation clinic within the Mental Health Services at the Landspitali - The National University Hospital of Iceland or self-referred from an advertisement to members of the Icelandic ADHD association, a national support organization. All participants were required to have a clinical diagnosis of ADHD and to be stable on prescribed ADHD medication for at least a month, i.e. stimulants (immediate- or extended-release methylphenidate and amphetamine sulphate), atomoxetine or bupropion. The participants were told to try and keep dosages unchanged during the whole study. Exclusion criteria included patients with severe mental illness, active drug abuse, verbal IQ estimated from clinical records to be below 85, no valid ADHD diagnosis or not prescribed/taking ADHD medication.

Out of the 92 referrals initially received, 38 were excluded on the following grounds: 13 were off-medication, nine with a questionable diagnosis and four misusing drugs/alcohol. A further seven declined to participate and five either did not show up for the intake interview or they could not be reached by phone or e-mail.

The remaining 54 participants were 34 women (mean age 34.1, SD = 10.9) and 20 men (mean age 33.5, SD = 12.4). Of the 54 participants 33 were self-referrals and 21 were referred by psychiatrists. All participants had been assessed and diagnosed with ADHD by mental health professionals with expertise in diagnosing ADHD using DSM-IV criteria. All medication was prescribed by psychiatrists. At baseline, 42 (77.8%) participants were receiving methyphenidate, 11 (20.4%) were receiving atomoxetine, 5 (9.3%) were receiving bupropion, and 1 (1.9%) was receiving amphetamine sulphate. Thirteen (24.1%) participants were receiving only one medication, 16 (29.6%) were receiving two medications and the remaining 25 (46.3%) were receiving three or more drugs. Participants were asked if they had some other mental/emotional problem and 35 (64.8%) reported depression, 20 (37%) reported some anxiety disorder, 12 (22.2%) reported a history of drug/alcohol abuse and nine (16.7%) reported some other psychiatric problem. Only eight (14.8%) did not report comorbid problems.

### Measures

#### Independent evaluation (IE)

The Kiddie-Schedule for Affective Disorders and Schizophrenia (K-SADS-PL) ADHD section, present and lifetime version [25] interview measures both ADHD symptoms and impairment on functioning (home, work and relationships) and has been modified for adults and translated into Icelandic. Magnusson et al. [26] found that the K-SADS was reliable and valid and had strong correlation with self-reported and informant rated ADHD symptoms. In the present study current symptoms were rated to measure symptom change. A total of 18 questions are rated on a 1-3 point scale from 1 = no symptoms or impairment, 2 = symptoms with moderate impairment, and 3 = symptoms indicating severe impairment in functioning. The minimum score on the K-SADS is 18 and 54 is the maximum score

The Clinical Global Impression (CGI; 27) is a single question where the clinician is asked to rate severity of illness on a 7 point scale (i.e., a score of 1 indicates not being ill and a score of 7 indicates being extremely ill) by comparing the patient to other patients with ADHD. The clinician's severity score is based on judgment regarding impairment in functioning, symptom severity and distress or coping and is supported by examples of these factors [27]. The CGI has shown to correlate well with other ADHD measures [28,29].

#### Self-report measures

The Barkley ADHD Current Symptoms Scale (BCS; [30]) corresponds to the DSM-IV diagnostic criteria of ADHD. Each item was scored on a 4-point Likert scale for frequency of symptoms experienced during the previous six months. Scores range between 0 and 27 for each of the two subscales (Inattention and Hyperactivity/Impulsivity) and 0 to 54 for the Total scale. The scale is reported to have good psychometric properties and correlates well with informants' ratings of symptoms and interview-based diagnoses in childhood and adulthood in an Icelandic sample [26].

The Beck Anxiety Inventory (BAI; [31]) is a 21-item scale designed to assess severity of anxiety symptoms. Items are scored on a 4 point Likert scale (0-3) where the respondent rates how much he or she has been bothered by various symptoms during the past week from not at all to severely.

The Beck Depression Inventory (BDI; [32]) is a 21-item scale where responders rate how they have been feeling during the past week on a 4 point Likert scale (0-3).

The R&R2 ADHD Training Evaluation Self-report Scale (RATE-S; [33]) provides four subscales: (1) ADHD symptoms; (2) Emotional Control; (3) Antisocial Behaviour; and (4) Social Functioning. The RATE-S scale has been shown to have good reliability and validity [11,34], Gudjonsson, Sigurdsson, Adalsteinsson & Young: The relationship between attention deficit hyperactivity disorder (ADHD) symptoms, mood instability, and self-reported offending, submitted).

### The Intervention

R&R2ADHD [33] is a 15 session manualised CBT intervention programme that was developed in 2007 for youths and adults with ADHD and antisocial behaviour. It is a revised edition of the 35-session Reasoning & Rehabilitation programme [35] that was originally developed as a prosocial competence training programme for use in correctional facilities and its feasibility and effectiveness are well supported in this population [36,37]. R&R2ADHD is a structured, manualised programme that aims to decrease impairment of core ADHD symptoms and improve social, problem solving, and organizational skills. It consists of five treatment modules (1) neurocognitive, e.g. learning strategies to improve attentional control, memory, impulse control and planning, (2) problem solving, e.g. developing skilled thinking, problem identification, consequential thinking, managing conflict and making choices, (3) emotional control, e.g. managing feelings of anger and anxiety, (4) pro-social skills, e.g. recognition of the thoughts and feeling of others, empathy, negotiation skills and conflict resolution, and (5) critical reasoning, e.g. evaluating options and effective behavioural skills.

The programme integrates group and individual treatment, the latter being achieved by group facilitators training 'coaches' who meet with the participant between sessions. The coaching role aims to support participants to transfer skills learned in the group into their daily lives. In the present study the coach role was fulfilled by psychology undergraduates. This programme was delivered according to a manual and the coaches also received directions through training and written guidelines. All R&R2ADHD facilitators had extensive experience in CBT and received training in delivering the programme.

### Procedure

The study was conducted in line with international guidelines, following ethical approval by the Icelandic Bioethics Committee on 01/09/2008, reference number 08-095-S1.

All 54 participants met with the first author for an intake interview when they gave informed consent. Of these 51 completed the self-reported baseline measures and 51 completed the baseline measures with the independent evaluator. The independent evaluators were psychiatrists who were blind to the treatment condition. They obtained demographic information and completed the K-SADS and CGI. Every attempt was made to maintain the blind evaluation as both independent evaluators and participants received repeated instructions to remind them to avoid disclosure of whether the participant was receiving R&R2ADHD group treatment or not.

An independent psychiatrist randomly allocated the participants to either the CBT/MED experimental condition (n = 27) or the TAU/MED control condition (n = 27). The CBT/MED condition received R&R2ADHD group therapy in addition to continued psychopharmacological treatment. The TAU/MED condition received psychopharmacological treatment only. At baseline no statistical difference (two-tailed) was found between the two conditions on dosage size of methylphenidate (t = 1.126, df = 40, p = .267), atomoxetine (t = .697, df = 9, p = .504), age (t = -.439, df = 52, p = .662), or sex (χ^2 ^= (1, N = 54) = 0.318, p = .573). No statistical differences were found on any of the outcome measures at baseline between the two conditions (p < .05).

**Figure 1:**
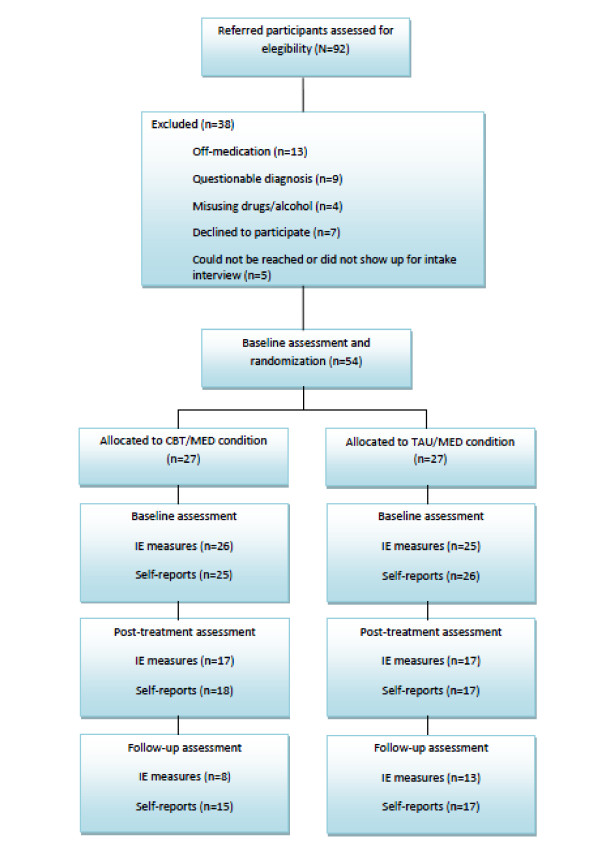
**Flowchart of patient participations**.

The participants in both conditions were not asked to refrain from engaging in other interventions during the study period. Information about other interventions was not collected and thus other treatments were not controlled for. Treatment integrity was ensured in two ways; first by adopting a structured manualised CBT programme and, second, via the independent observation of a sample of sessions by a practitioner who monitored adherence to the manualised treatment protocol. Participants in the CBT condition received 15 R&R2ADHD sessions twice weekly, each lasting 90 minutes. Three groups were run in total and coaches met with the participants once a week for 30 minutes to review sessions and help with homework. Participants were re-assessed using the same measures at Time 2 (end of treatment) and Time 3 (three month follow up). The timing of the evaluation assessments was the same for the CBT/MED and TAU/MED conditions. A log of group attendance, and reasons for non-attendance were recorded each session. Figure 1 presents a flowchart of patient participation.

### Statistical analysis

Unadjusted mean scores and standard deviations on each of the outcome measures are provided for the CBT/MED and TAU/MED conditions for the three assessment periods - Time 1, Time 2 and Time 3 (see Table 1). Differences between the two conditions on the outcome measures were not statistically significant at baseline. Nevertheless, in order to reduce error variance an analysis of covariance (ANCOVA) was calculated for each of the dependent variables measuring differences between the conditions in time. The baseline scores therefore served as covariates and scores at Time 2 and Time 3 served as dependent variables. Thus intention to treat analysis (ITT) was conducted. Missing values were not imputed because the ANCOVA calculates outcome whilst adjusting for all baseline data. Between group effect sizes for the outcome assessments were measured using Cohen's d using unadjusted means for the dependent variables and SD pooled for unequal group sizes. Fischer's exact test was used to compare proportions of medication changes. Since this study follows an ITT protocol, statistical analysis of the outcome variables were completed for all participants regardless of medication changes.

**Table 1:** Means and standard deviations and between group effect sizes (Cohen's d) at outcome

	CBT/MED			TAU/MED				
**Outcome measures**	**Baseline** **Mean(SD)**	**End of treatment** **Mean(SD)**	**Three month follow-up** **Mean(SD)**	**Baseline** **Mean(SD)**	**End of treatment** **Mean(SD)**	**Three month follow-up** **Mean(SD)**	**End of treatment** **Cohen's *d***	**Follow-up** **Cohen's *d***

CGI	4.00(.85) n = 26	3.18(1.07) n = 17	3.00(.76) n = 8	4.24(1.05) n = 25	3.88(.70) n = 17	4.08(.86) n = 13	n.s.	1.31*

K-SADS ADHD	40.02(5.35) n = 26	29.88(7.23) n = 17	31.70(4.33) n = 8	38.16(8.14) n = 25	35.94(4.08) n = 17	37.08(4.72) n = 13	1.03**	1.17*

BCS inattention	15.84(6.28) n = 25	10.17(4.44) n = 18	9.76(5.62) n = 15	16.54(6.84) n = 26	14.71(5.19) n = 17	16.24(5.66) n = 17	0.94*	1.15**

BCS hyperactivity/ impulsivity	12.88(5.00) n = 25	7.06(4.41) n = 18	5.94(4.12) n = 15	9.75(6.17) n = 26	8.76(6.22) n = 17	8.76(5.43) n = 17	0.32*	0.58**

BCS Total score	28.72(10.21) n = 25	17.22(7.62) n = 18	15.70(8.74) n = 15	26.29(11.07) n = 26	23.47(8.80) n = 17	25.00(8.54) n = 17	0.76**	1.08***

BAI Anxiety	13.43(8.67) n = 25	11.00(10.61) n = 18	7.25(5.91) n = 15	14.06(7.73) n = 26	15.29(10.72) n = 17	12.89(7.50) n = 17	n.s.	0.83*

BDI Depression	11.60(8.71) n = 25	7.22(6.84) n = 18	5.00(5.77) n = 15	16.09(10.61) n = 26	15.41(9.64) n = 17	15.43(9.25) n = 17	n.s.	1.32*

RATE ADHD symptoms	41.76(11.73) n = 25	34.88(9.42) n = 17	29.12(10.94) n = 14	40.31(13.95) n = 26	41.12(10.86) n = 17	42.00(12.67) n = 17	n.s.	1.08**

RATE Emotional Control	33.24(14.63) n = 25	27.47(11.01) n = 17	21.50(9.59) n = 14	35.73(13.17) n = 26	33.16(12.84) n = 17	36.29(15.58) n = 17	n.s.	1.12*

RATE Antisocial Scale	11.70(4.36) n = 25	9.12(1.41) n = 17	9.00(1.75) n = 14	13.27(7.24) n = 26	10.76(2.39) n = 17	12.06(4.37) n = 17	0.84*	0.89*

RATE Social Functioning	28.52(7.53) n = 25	26.76(9.25) n = 17	24.29(8.07) n = 14	32.46(10.31) n = 26	36.47(10.76) n = 17	36.41(10.93) n = 17	n.s.	1.24**

RATE total score	115.22(29.17) n = 25	98.24(23.14) n = 17	82.20(25.10) n = 14	121.77(30.69) n = 26	121.35(24.08) n = 17	126.76(31.96) n = 17	n.s.	1.46***

Significant results *(*p *< .05) ** (p < .01) *** (p < .001); n.s. = no between group significance.

## Results

### Completion Rate

Of the 27 participants who started the CBT treatment, 20 participants completed, giving a completion rate of 74%. Four dropped out during the treatment phase without explanation, one due to moving out of the area, one due to illness in the family and one had to stop medication due to pregnancy. The dropout rate of 6 (22.2%) was similar for participants in the TAU/MED condition (i.e. they did not attend the end of treatment assessment). Two participants in the CBT treatment condition and four participants in the control condition did not complete all of the end of treatment assessments. A further three participants in the CBT treatment condition but no participants in the control condition did not complete the follow-up assessments.

A total of 35 participants completed self-reported questionnaires at the end of treatment and 32 at three month follow up; 34 participants attended the independent evaluation at the end of treatment and 21 at three month follow-up. To test for possible baseline differences between completers and non-completers a comparison was made on baseline IE measures between those who completed the follow-up measures and those who attended the baseline measures but did not complete all the post assessments (two tailed). For the CBT/MED condition there was no statistical difference at baseline between completers (n = 8) and non-completers (n = 18) on the CGI (t = .493, df = 24, p = .626) or on the K-SADS (t = .720, df = 24, p = .479). The same results were found for the TAU/MED condition where no statistical difference was found between completers (n = 13) and non-completers (n = 12) on baseline measures of CGI (t = .419, df = 23, p = .679) or K-SADS (t = .480, df = 23, p = .636).

### Medication changes

At baseline, methylphenidate dosages ranged between 18-180 mg, with a mean dosage of 60.5 mg. By the end of treatment, dosages had been increased for two participants in each condition and decreased for one participant in each condition. The dosage range for methylphenidate was 36-162 mg, with a mean dosage of 62.5 mg. At three-month follow-up dosages had been increased for one participant in each condition and decreased for two in the CBT/MED condition and one in the TAU/MED condition. The dosage range of methylphenidate at follow-up was 36-108 mg, with a mean dosage of 59.4 mg. Fischer's exact test revealed that there were no significant differences in proportions of medication change between the two conditions either at the end of treatment (P = .619) or at three month follow-up (P = .473). Table 1 presents the unadjusted means and standard deviations for each outcome measure at baseline, at the end of treatment and at three month follow up, for the experimental (CBT/MED) and control (TAU/MED) conditions. It also gives the effect sizes (Cohen's *d*) of the mean difference between the two conditions for the end of treatment and three-month follow-up assessments. Adverse events were recorded during the trial and one participant in the CBT/MED condition reported severe distress at the end of treatment due to changes in personal circumstances. This participant then received individual treatment and was not assessed at follow-up.

### Effectiveness

#### Independent evaluators' outcome measures (IE)

**Figure 2:**
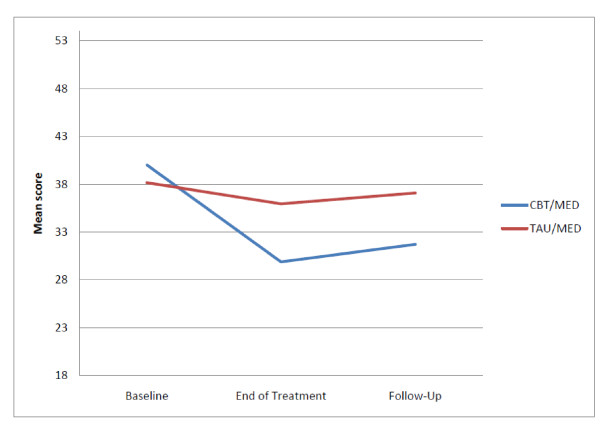
**Independent evaluator rated changes in unadjusted means on the K-SADS ADHD measure**.

After adjusting for baseline means the CBT/MED condition had significantly lower IE ratings than the TAU/MED condition on the K-SADS ADHD measure at the end of treatment (*F*(1,31) = 11.02, *p *< .01) with a large effect size. At three month follow-up a significant difference was maintained where the CBT/MED condition had lower IE ratings than the TAU/MED condition (*F*(1,18) = 7.60, *p *< .05) and the effect size remained large (see Figure 2).

On the CGI no significant difference was found between conditions at the end of treatment (*p = .06*) but the CBT/MED condition had significantly lower IE ratings at follow-up (*F*(1,18) = 9.16, *p *< .05) with a large effect size.

#### Self-report outcome measures

**Figure 3:**
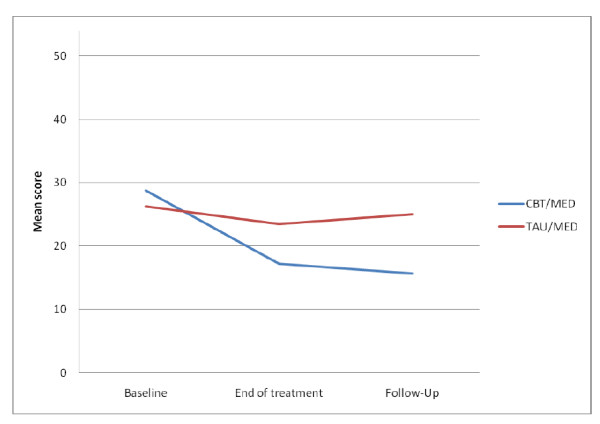
**Self-reported changes in unadjusted means on the Barkley ADHD Current Symptom Scale**.

After adjusting for baseline means the participants in the CBT/MED condition had significantly lower scores on the inattention scale of the BCS than those in the TAU/MED condition at the end of treatment (*F*(1,32) = 8.73, *p *< .05) and at three month follow-up (*F*(1,29) = 10.70, *p *< .01) with large effects sizes. The participants in the CBT/MED condition also scored lower on symptoms of hyperactivity/impulsivity on the BCS both at the end of treatment (*F*(1,32) = 7.27, *p *< .05) and at three month follow-up (*F*(1,29) = 20.30, *p *< .001) with small and medium effect sizes, respectively. On the total BCS score the participants in the CBT/MED condition scored significantly lower than those in the TAU/MED condition at the end of treatment (*F*(1,32) = 10.45, *p *< .01) and at follow-up (*F*(1,29) = 17.36, *p *< .001) with medium and large effect sizes, respectively (see Figure 3).

After adjusting for baseline means no significant difference was found on anxiety scores on the BAI between the two conditions at end of treatment (*p *= .46). The participants in the CBT/MED condition showed however significant improvement at follow-up compared with those in the TAU/MED condition (*F*(1,29) = 4,61, *p *< .05) with a large effect size. On the BDI no significant difference was found at the end of treatment (*p *= .052) but the CBT/MED condition showed significant improvement compared with the TAU/MED condition at follow-up (*F*(1,29) = 5.86, *p *< .05) with a large effect size.

With respect to the RATE-S Scales, no significant difference was found between the two conditions at the end of treatment on the Total RATE-S score (*p *= .07) but the CBT/MED condition scored significantly lower than the TAU/MED condition at follow-up (*F*(1,28) = 14.77, *p *< .001) with a large effect size. The same effect was found for the ADHD, Emotional Control and Social Functioning Scales. No significant difference was found between the two conditions at the end of treatment on the ADHD Scale (*p = *.16) but the CBT/MED condition scored significantly lower than the TAU/MED condition at three month follow-up (*F*(1,28) = 11.83, *p *< .01) with a large effect size. No significant difference was found between the two conditions at the end of treatment on the Emotional Control Scale (*p = .48*) but at follow-up the CBT/MED condition showed significant improvement compared with the TAU/MED condition (*F*(1,28) = 6.35, *p *< .05) with a large effect size. On the Social Functioning Scale no significant difference was found between the two conditions at the end of treatment (*p *= .09) but the CBT/MED condition showed significant improvement compared with the TAU/MED condition at follow-up (*F*(1,28) = 10.88, *p *< .01) with a large effect size. On the Antisocial Scale, the CBT/MED condition showed significant improvement compared with the TAU/MED condition at the end of treatment (*F*(1,31) = 4.75, *p *< .05) with a large effect size. This difference was maintained at follow-up (F(1,29) = 7.28, *p *< .05) with a large effect size.

## Discussion

Two important findings arise from the results. As hypothesized there was a significant effect for improvement in core ADHD symptoms at the end of treatment. Secondly, large effects were found for treating ADHD symptoms and comorbid problems at follow up. The exception is the BCS hyperactivity/impulsivity scale where the effect sizes were small to medium. It is however evident from the present findings that in spite of receiving medication for ADHD, the participants were experiencing significant residual symptoms which were successfully and further improved by the CBT intervention. Safren and colleagues [16,17] also reported that combined treatments have better outcomes than medication alone in treating ADHD symptoms, depression and anxiety.

Antisocial behaviour also improved at the end of treatment and at follow-up with a large effect. This is noteworthy since participants' baseline scores for antisocial behaviour were relatively low for both conditions indicating the importance of the prosocial training component of R&R2ADHD. Given the reported high rates of comorbid antisocial problems in adult ADHD [2-4], it seems important to include a prosocial competence component to CBT interventions when treating people with ADHD. The present study illustrates that even in participants who have not been referred for antisocial behaviour, a more positive prosocial outcome can be achieved. Alternatively, antisocial participants need to be screened out of CBT interventions that aim primarily to target core ADHD symptoms of attention, impulsivity, planning and organization deficits, else it is possible that improvement in functioning in these domains may be applied to improve antisocial skills.

Significant and large treatment effects were noted on all the self- reported measures when followed up three months later. This was supported by the independent evaluations of ADHD symptoms and global functioning which had large effect sizes. For the ADHD symptoms, effect sizes were even greater at follow up than at the end of treatment. Thus the R&R2ADHD programme was highly effective in treating ADHD symptoms and common comorbid problems of anxiety, depression, antisocial behaviour and social functioning. Improvements in comorbid problems were partly significant immediately following the end of treatment phase but significantly and further improved during the follow-up period. It is likely that those who completed the CBT intervention continued to use the strategies learned in sessions after they finished treatment and therefore the treatment effect persisted and became greater over time.

The present study shows that the RATE-S Scales, which are provided with the programme, are useful dynamic measures of change over time as people symptomatic for ADHD learn to cope better with the emotional instability associated with their symptoms. This is in line with other studies using the RATE-S [11,34]. It also shows that R&R2ADHD is an effective intervention for ADHD adults attending psychiatric community services and participants reported to facilitators that they enjoyed attending the programme. As a structured manualized programme, R&R2ADHD facilitates consistency in delivery across different populations and settings and maximises programme integrity. Thus the benefits of R&R2ADHD are multifaceted and the combination of psychopharmacological and CBT treatments may add to and improve pharmacological interventions. This is likely to be further enhanced by the integration of group sessions and individual coaching sessions as a model for programme delivery as this model provides a structured support for the transference of skills into daily life.

The strengths of the current study are its RCT design and the independent outcome measures used in addition to self-report measures. There was a modest drop-out rate for this kind of a study and the drop-out rate was comparable between both conditions. The main limitations of the study are the small numbers of participants and the difficulties to obtain outcome measures for all participants at the end of treatment and at follow-up. The attrition rate for outcome measures is a common problem with this kind of research [38].

A second limitation is that we were unable to control for change in medication as study participants remained under the care of their individual treating psychiatrists. Although there were some changes in medication, these did not significantly differ between the two conditions. Furthermore, we did not control for the possibility that the TAU/MED condition were receiving some other non-pharmacological interventions.

A further limitation is that the participants in the CBT/MED condition received more attention than the TAU/MED participants during the treatment phase and therefore nonspecific placebo effects could limit the results. However, most changes occurred during the period between the end of treatment and three month follow-up and both conditions did not receive any contact during this period.

## Conclusions

The results give further support for the growing evidence that CBT increases the effect of psychopharmacological treatment in reducing ADHD symptoms and comorbid problems, and demonstrating improvements in functions associated with impairment. These findings support the recommendations of international guidelines for a comprehensive treatment package that includes psychological and psychopharmacological treatments for adults with ADHD.

## Abbreviations

ADHD: Attention Deficit Hyperactivity Disorder, R&R2ADHD: Reasoning and Rehabilitation for ADHD Youths and Adults, CBT: Cognitive Behavioural Treatment, RCT: Randomized Controlled Trial, CBT/MED: group condition receiving CBT and medication, TAU/MED: control condition receiving 'treatment as usual' and medication, KSADS ADHD: Kiddie-Schedule for Affective Disorders and Schizophrenia, ADHD Scale, CGI: Clinical Global Impression, BCS: Barkley ADHD Current Symptoms Scale, BAI: Beck Anxiety Inventory, BDI: Beck Depression Inventory, IE: Independent Evaluator.

## Competing interests

BE, JFS, GB, EE & HO declare that they have no competing interests. SY has been a consultant for Janssen-Cilag, Eli-Lilly and Shire. She has given educational talks at meetings sponsored by Janssen-Cilag, Shire, Novatis, Eli-Lilly and Flynn-Pharma and has received research grants from Janssen-Cilag, Eli-Lilly and Shire. SY is a consultant for the Cognitive Centre of Canada and is co-author of 'R&R2 for ADHD Youths and Adults'. GG has been a consultant for Eli-Lilly and given educational talks at meetings sponsored by Janssen-Cilag and Shire.

## Authors' contributions

BE, JFS and GB secured financial support for the study. SY provided training in R&R2ADHD. BE and EE carried out the R&RADHD treatment and BE, JFS & GG handled the statistical procedures. GB and HO served as the independent evaluators. JFS, GG and SY supervised BE and EE. All authors contributed to the study design and writing the manuscript. All authors have read and approved the manuscript.

## Pre-publication history

The pre-publication history for this paper can be accessed here:

http://www.biomedcentral.com/1471-244X/11/116/prepub
